# Phase I safety study of 0.5% PRO 2000 vaginal Gel among HIV un-infected women in Pune, India

**DOI:** 10.1186/1742-6405-3-4

**Published:** 2006-02-20

**Authors:** Joshi Smita, Dutta Soma, Bell Beverly, Profy Albert, Kuruc JoAnn, Gai Fang, Cianciola Missy, Soto-Torres Lydia, Panchanadikar Anjali, Risbud Arun, Mehendale Sanjay, Reynolds Steven J, the HIV Prevention Trial Network (HPTN) 047 Protocol Team

**Affiliations:** 1 National AIDS Research Institute, India; 2 Family Health International, USA; 3 Indevus Pharmaceuticals, Inc., USA; 4 Fred Hutchinson Cancer Research Center-SCHARP, USA; 5 The National Institute of Allergy and Infectious Diseases, National Institutes of Health; 6 The National Institute of Allergy and Infectious Diseases, National Institutes of Health and Johns Hopkins University School of Medicine, USA

## Abstract

**Background:**

The objective of this study was to evaluate the safety of twice daily, intra-vaginal use of 0.5% PRO 2000 Gel for fourteen days in HIV un-infected women at lower as well as higher risk for HIV acquisition, in Pune, India.

**Methods:**

Forty-two eligible volunteers (30 low-risk and 12 high-risk) were given 0.5% PRO 2000 Gel for intra-vaginal application twice daily for 14 consecutive days.

**Results:**

Twenty-four participants (57%, 95% CI 41%–72%) experienced at least one adverse event (AE) judged to be possibly related to the product use. There were 17 (40%, 95% CI 26%–57%) mild AEs and 7 (17%, 95% CI 7%–31%) moderate AEs. There were no serious adverse events and no AEs judged probably or definitely related to product use. Genitourinary discomfort was reported by 2/30 (6.67%) participants in the low-risk cohort as compared to 4/12 (33.3%) women in the high-risk cohort (p = 0.03). Intermenstrual bleeding was reported in 2/30 (6.7%, 95% CI 1.0–22.1) women from the low risk cohort and 3/12 (25%, 95% CI 5.5–57.2) women from the high-risk cohort. One participant showed mild elevation of blood gamma glutamyl transferase and two showed mild elevations in total bilirubin. None of the participants showed detectable PRO 2000 in their blood after 14 days of product use.

**Conclusion:**

0.5% PRO 2000 Gel appeared to be safe when used twice-daily by sexually active HIV-uninfected women from Pune, India. Although genitourinary discomfort and metrorrhagia were more common in the high-risk cohort, ongoing Phase II/IIb trial would provide data for generalization of this finding.

## Introduction

Vaginal microbicides are products designed to prevent sexual transmission of HIV and other sexually transmitted pathogens[1]. They may help women in situations where negotiation of male condom use is not possible. PRO 2000 Gel is an investigational vaginal microbicide based on a synthetic naphthalene sulphonate polymer that has been shown to be active against HIV, herpes simplex virus type 2 (HSV-2) and other pathogens causing sexually transmitted infections (STI) in vitro [2-5]. The vaginal gel formulation demonstrated protection in animal models for HIV, HSV-2 and Neisseria gonorrhoeae transmission [6-8] at strengths as low as 0.5%.

Phase I clinical trials to assess the safety, tolerance and acceptability of PRO 2000 Gel among women in the reproductive-age group have been conducted in Europe, the United States (U.S.) and Africa[9,10]. In addition, trials to assess the safety and acceptability of repeated penile exposure to PRO 2000 Gel for 7 consecutive days among HIV un-infected and infected men have been conducted in the U.S.[11]. None of the previous studies were conducted in Asia. The International Working Group for Microbicides recommends evaluation of candidate vaginal microbicides for safety, tolerance and acceptability in populations with different characteristics[12]. The objective of this study was to evaluate the safety and acceptability of 0.5% PRO 2000 Gel among sexually active, HIV un-infected, low-risk and high-risk women from Pune, India and to assess if transient findings of cervico-vaginal disruption reported in pig-tailed macaques treated for 4 days with PRO 2000 Gel (2% and 4% strengths) [13] were predictive of human toxicity.

## Materials and methods

This collaborative study between National AIDS Research Institute (NARI), Pune, India and Johns Hopkins University School of Medicine, Baltimore, USA was funded by the HIV Prevention Trials Network (HPTN). The Ethics Committee of NARI, the Drug Controller General of India and the Western Institutional Review Board of Johns Hopkins University approved the study protocol. The protocol was also submitted to the United States Food and Drug Administration. The study was conducted at our clinic located in Hirabai Cowasji Jehangir Medical Research Institute, Jehangir Hospital and Medical Center, Pune, India.

The study was conducted between July 2003 and March 2004 and HIV uninfected sexually active, low-risk as well as high risk women were screened for possible enrollment. Women were classified as high-risk if they or their male partner had a documented Sexually Transmitted Infection (STI) within three months of screening. Women between 18 and 45 years of age, HIV-negative by licensed EIA, having a regular menstrual cycle with a minimum of 21 days between menses, not having any Grade III or higher hematological, liver and renal abnormality, having a normal Pap smear at screening, or within the 3 months prior to screening and those having a single male sexual partner were enrolled in the study. Women who were pregnant, lactating or using any intra-uterine contraceptive device were not eligible to participate in the study. The National Institute of Allergy and Infectious Diseases Table for the Grading of Adult Adverse Experiences (AE) was used to characterize the severity of Adverse Events occurring in the study.

### Clinical, laboratory procedures and end points

All women provided written informed consent prior to study participation. Women were screened for their eligibility to participate in the study and if eligible, were enrolled within 30 days of screening. At screening visits, demographic data were collected using structured questionnaires, pelvic examinations were completed and blood samples collected for HIV testing, Rapid plasma reagin (RPR) with confirmation by Treponema pallidum haemagglutination (TPHA), complete blood count, biochemistry and coagulation testing. Participants were requested to bring their male partners to provide consent for study participation and HIV testing. Female and male participants diagnosed with any STI were given treatment as per the 2001 World Health Organization (WHO) guidelines for the management of sexually transmitted infections[14]. Male partners were informed to report to the study clinic if they had any genital symptom due to product exposure.

Women were requested to return for an enrollment visit within two to seven days after menstruation, if the results of all screening tests were within normal limits. Colposcopy was performed at the enrollment visit (considered study day 0) and at scheduled follow up visits at day 2 and day 14 (after completing 14 days' of product use). Additional colposcopic evaluation was done if any clinically detectable abnormality was seen on naked eye examination at day 7 or during any unscheduled visit requiring pelvic examination. The colposcopic evaluation was performed according to the CONRAD/WHO Manual for the Standardization of Colposcopy for the Evaluation of Vaginal Products, Update 2000[15]. The study staff completed a structured questionnaire whenever an inter-menstrual bleeding (IMB) episode was observed clinically or reported by the study participants. The study endpoints were macroscopic evidence of cervico-vaginal ulceration, abrasion, severe erythema or edema as determined by speculum-assisted colposcopic examination or completion of product use twice-daily for 14 days.

Participants who were eligible for enrollment based on pelvic examination, laboratory reports and colposcopy were given the product kits containing single dose tubes of 0.5% PRO 2000 Gel and an equal number of calibrated polyethylene vaginal applicators. They were asked to apply a 2 gm dose of the product intra-vaginally twice daily (preferably morning and bedtime) for two days before returning for a follow up clinical and colposcopic examination on day 2. Product use was continued for another 5 days and a clinical examination was performed on day 7. Product use was continued for 7 more days and the final clinical examination and colposcopy were performed on day 14.

During pelvic examination, specimens were collected for Pap smear (only at screening), for vaginal pH test using colorpHast^® ^Indicator strip (0–6) from the vaginal wall, for wet mount to assess for the presence of trichomoniasis, candidiasis or bacterial vaginosis from anterior and lateral fornix, dried smear from the lateral vaginal wall for Nugent's scoring[16] and urine examination by polymerase chain reaction (PCR) for gonorrhoea and chlamydia (only at screening and if indicated at other visits).

Criteria for discontinuation from the product use were clinical diagnosis of any associated significant side effect, non-adherence to study protocol or unwillingness of the participant to continue further product use. Statistical analysis was performed using the SAS^® ^and StatXact statistical software packages.

### Acceptability assessment

Acceptability of the product was assessed after 14 days of product use on structured questionnaires in case of all women and through focus group discussions conducted among women and their male partners.

## Results

Seventy women were screened for possible enrollment in the study, of which 42 were found eligible and were enrolled in the study. Thirty participants were enrolled in lower risk and 12 in the higher risk cohort. Baseline demographics were similar in both low and high-risk cohorts. Mean age for the enrolled participants was 30.2 years (range 21–42 years). All participants were married and living with their husbands, and were literate enough to complete a daily diary; 40 (95%) had studied above 5^th ^grade. The majority of them had undergone tubal ligation (33/42, 78.6%), whereas other methods of contraception being used were oral contraceptive pills (2/42, 4.8%), male condoms (2/42, 4.8%), and vasectomy in case of one couple. The remaining 4/42 (9.5%) of couples were not using any birth control method.

Of the forty-two participants enrolled, forty-one participants completed the study as planned, and all were considered adherent to product use (defined as 14 consecutive days of twice daily product use, allowing one or two days of additional product usage if missed during the 14 days' of product use). One participant was enrolled in the study and was dispensed the study product. However, during the interview with the clinician, the participant reported irregular vaginal bleeding and hence terminated after enrollment but before any product was used. Since she was enrolled in the study, and then terminated, she has been included in the analysis as per the "intent to treat" analysis.

The median sexual activity reported by the study participants was 3.3 acts per week over the two-week study period. Of the 42 enrolled participants, 24 (57%, 95% CI 41%–72%) experienced at least one AE judged to be possibly related to product use. All AEs were mild or moderate in severity. Table 1 shows the incidences of self-reported symptoms, pelvic examination/colposcopic findings and laboratory AEs observed among the study participants and judged possibly related to the product use. No serious adverse events were reported and no AEs were judged to be probably or definitely related to product use. No AEs were reported by the participants' male partners.

**Table 1:** Summary of self-reported symptoms, Colposcopic findings and Laboratory Adverse Experiences

**Self-Reported Adverse Experiences Judged Possibly Related to Study Product**				
**Adverse event**	**Low-risk cohort ** **N = 30** ** N (Mild, Moderate)**	**High-risk cohort ** **N = 12** ** N (Mild, Moderate)**	**Total N = 42 (%)**	**Outcome**

Abdominal pain	6 (5, 1)	3 (1, 3)	9 (21.4%)	Resolved
Back pain	3 (3, 0)	1 (1, 0)	4 (9.5%)	Resolved
Dyspareunia	1 (1, 0)	0	1 (2.4%)	Resolved
Dysuria	1 (1, 0)	0	1 (2.4%)	Resolved
Genito-urinary discomfort*	2 (2, 0)	4 (2, 2)	6 (14.3%)	Resolved

**Colposcopic and Laboratory Adverse Experiences Judged Possibly Related to Study Product**				

**Adverse event**	**Low-risk cohort (N = 30)** ** N (Mild, Moderate)**	**High-risk cohort (N = 12)** ** N (Mild, Moderate)**	**Total N = 42 (%)**	**Outcome**

Candidiasis	2 (0, 2)	0	2 (4.8%)	Resolved
Cervical Polyp	2 (2, 0)	0	2 (4.8%)	Continuing**
Intermenstrual bleeding (IMB)	2 (2, 0)	2 (2, 0)	4 (9.5%)	Resolved
Abrasion				
Cervical	0	1 (1, 0)	1 (2.4%)	Resolved
Vaginal	1 (1, 0)	0	1 (2.4%)	Resolved
Increased GGT	0	1 (1, 0)	1 (2.4%)	Resolved at a follow up after 10 days
Hyperbilirubinaemia	2 (2, 0)	0	2 (4.8%)	Continuing**

Participants with moderate adverse events were provided symptomatic treatment.

* "Genitor-urinary discomfort" includes "genital burning sensation" & "genital pruritus". ** The adverse events were observed on day 14 at the time of study termination and participants did not come for follow up visit.

None of the colposcopic examinations showed evidence of ulceration, severe edema, or severe erythema of the vulvovaginal epithelium or cervical mucosa. A mild cervico-vaginal abrasion was observed in two participants at the scheduled Day-2 pelvic examination. Speculum trauma was considered a possible cause of each abrasion, and each resolved within 2–3 days without discontinuation of PRO 2000 Gel use.

Genito-urinary discomfort which includes "genital burning sensation" & "genital pruritus" were reported in 2/30 (6.67%, 95% CI 1.0–22.1) women in the low risk cohort as compared to 4/12 (33.3%, 95% CI 9.4–65.6) women in the high-risk cohort (P = 0.03). Inter-menstrual bleeding episodes (IMB) were reported in 2/30 (6.7%, 95% CI 1.0–22.1) women from the low risk cohort and 3/12 (25%, 95% CI 5.5–57.2) from the high-risk cohort, though in one case the event was judged unrelated to study product use. Of the five participants who were diagnosed with IMB, one participant was on oral contraceptive pills for the past three and a half years and the other four had undergone tubal ligation.

Of the 42 enrolled participants, 11 (26.2%, 95% CI 14%–42%) developed asymptomatic candidiasis at follow up visits and two participants (4.8%, 95% CI 0.6%–16.2%) developed symptomatic candidiasis on day 7 requiring temporary stoppage (3 to 5 days) of the product use and oral medication with fluconazole (150 mg single dose) and local application of clotrimazole-betamethasone cream for genital itching.

The mean vaginal pH at enrollment as well as after 14 days of product use was 4.5 and none of the participants developed an alkaline pH. When the mean microflora scores of the Lactobacillus morphotypes were evaluated as per the Nugent's criteria[16], there was reduction in the normal flora from 30/42 (71%) to 18/42 (46%) participants after 14 days of product use however, the difference was not statistically significant. Though not significant the prevalence of BV decreased from 12% at enrollment to 10% at Day 14 (as per Nugent's criteria). At the Day 14 visit, one participant (2.4%, 95% CI 0.1%–12.6%) showed mild elevation of blood gamma glutamyl transferase (Participant's value 40 units/liter, Normal range: 5–36 U/liter, Grade I of DAIDS Toxicity Table 1.25-2.5 × UPN) and two (4.8%, 95% CI 0.6%–16.2%) showed mild elevation of blood bilirubin levels (Participants' value 1.1 and 1.6 respectively, Normal range: 0.2–1 mg/dl, Grade I of DAIDS Toxicity table: >1.0 to 1.5 × ULN). The increased level of blood gamma glutamyl transferase returned to normal at a follow up visit after 10 days, however, the participants with raised bilirubin did not return for a follow up visit. None of the participants showed clinically significant changes in hematology parameters, other liver or renal function tests, or coagulation times. None had detectable PRO 2000 in plasma after 14 days of the product use.

The product was found to be generally acceptable by the study participants. Almost all the participants who used the product (40/41, 97.6%, 95% CI 87.1%–100%) expressed willingness to use the product in future if they felt at risk of HIV infection.

## Discussion

0.5% PRO 2000 Gel was observed to be safe and well tolerated in a twice-daily dosage schedule among the low risk as well as the high-risk women from Pune, India. All AEs were mild to moderate and transient. PRO 2000 Gel is one of the five candidate microbicides currently entering the Phase II/IIb efficacy trials[17] and this study has generated additional safety data.

The most common symptoms experienced by the study participants were abdominal discomfort and genito-urinary discomfort. Genitourinary discomfort and IMB were more common in the high-risk cohort as compared to the low-risk cohort, and this association needs to be studied further in the effectiveness trials where the product will be used by larger number of women for longer duration.

We could not determine the reason for elevated liver function tests in 3/42 (7.1%) of the study participants since there was no correlation with the plasma PRO 2000 levels and it is possible that these changes could have occurred because of reasons not related to product use. Study discontinuation was not warranted due to any of the AEs. In the absence of a placebo control arm we could not determine whether the reported AEs were related to PRO 2000 Gel use. It has been previously reported that women who were intensively monitored and were not using vaginal products other than tampons were more likely to report high levels of irritative symptoms[18,19]. In earlier Phase I studies[9,10], scheduled colposcopic assessments were not performed after two days of product use as in the present study. We did not observe any cervico-vaginal epithelial disruption among the study participants after two days of product use contrary to observations in pig-tailed macaques after treatment with 2% and 4% PRO 2000 Gel[13].

The IMB episodes experienced by participants in our study were mild, transient and well tolerated. None of the participants reported prior history of IMB episode. IMB was reported in previous Phase I clinical trials of the same product[9,10].

Since microbicides will act in a complex environment and an acidic pH may inactivate HIV and other sexually transmitted pathogens[20,21], the effects of PRO 2000 Gel on vaginal pH and microflora were also assessed. It was observed that the acidic pH was maintained in all the participants. Although there was a trend toward reduction in the normal vaginal microflora between enrollment and 14 days of product use, the difference was not statistically significant. We also observed reduction in incidence of bacterial vaginosis after 14 days of product use. Efficacy trials with larger sample size would provide definitive answers for the effect of PRO 2000 on normal vaginal flora and bacterial vaginosis. Acceptability of the product was not affected by the AE experiences and willingness to use the product in future was high.

To date, there have been relatively few microbicide studies conducted in India[22], despite the presence of HIV infection for more than 19 years. Epidemiological studies in India have reported higher rates of HIV infection among married, monogamous women [23-25] and microbicides research has to be accelerated in India through commitment at the policy and program management levels and by increasing involvement of institutions and non-governmental organizations. The necessary infrastructure such as clinical expertise, laboratory support and data management facilities needed to conduct microbicide studies already exist in India and should be utilized.

## Authors' contributions

JS contributed to study design, data collection, data interpretation and manuscript preparation. DS contributed to data collection and manuscript preparation. BB and KJ were involved in study design, conduct and manuscript writing. PA and MS contributed to study design and critical revising for important intellectual content. GF and CM were involved in data analysis, interpretation and manuscript writing. SL was involved in study design, conduct and manuscript writing. PA and RA were involved in laboratory procedures and manuscript writing. RSJ was involved in study design, conduct and critical review of the manuscript for its intellectual contents.
